# The Influence of Laparoscopic Sleeve Gastrectomy on Body Composition and Fat Distribution in Obese Caucasian Men and Women

**DOI:** 10.1007/s11695-020-04766-z

**Published:** 2020-06-16

**Authors:** Marek Tałałaj, Agata Bogołowska-Stieblich, Michał Wąsowski, Artur Binda, Paweł Jaworski, Małgorzata Wrzosek, Wiesław Tarnowski

**Affiliations:** 1 Department of Geriatrics, Internal Medicine and Metabolic Bone Diseases, Centre of Postgraduate Medical Education, Orłowski Hospital, Czerniakowska 231, 00-416 Warsaw, Poland; 2 Department of General, Oncological and Digestive Tract Surgery, Centre of Postgraduate Medical Education, Orłowski Hospital, Czerniakowska 231, 00-416 Warsaw, Poland; 3 Department of Biochemistry and Pharmacogenomics, Faculty of Pharmacy, Medical University of Warsaw, Banacha 1, 02-097 Warsaw, Poland; 4 Laboratory of Biochemistry and Clinical Chemistry at the Centre for Preclinical Research, Medical University of Warsaw, Banacha 1b, 02-097 Warsaw, Poland

**Keywords:** Obesity surgery, Laparoscopic sleeve gastrectomy, Weight loss, Body composition, Adipose tissue, DXA

## Abstract

**Background:**

The aim of the study was to assess changes in body composition in patients subjected to laparoscopic sleeve gastrectomy (LSG).

**Methods:**

Changes in body composition following LSG were determined in a group of 155 patients with obesity (117 women and 38 men), with dual-energy X-ray absorptiometry (DXA). Whole body fat mass (FM) and lean body mass (LBM) were determined, and abdominal fat mass (AbdF) was assessed within the region extending from the top of the pubic bone up to the line between 12th thoracic and 1st lumbar vertebras.

**Results:**

Over the period of 12 months following LSG, body mass index decreased by 28.2 ± 9.0% (*p* < 0.001). The reduction of body weight by 35.4 ± 12.6 kg (*p* < 0.001) was the result of a decrease in FM by 23.9 ± 8.9 kg (*p* < 0.001) and LBM by 10.5 ± 3.8 kg (*p* < 0.001). AbdF decreased from 13.2 ± 3.1 to 8.2 ± 2.7 kg (*p* < 0.001), but abdominal fat to total fat mass ratio increased from 24.9 ± 4.7 to 28.0 ± 5.8% (*p* < 0.001). The loss of AbdF was more pronounced in men than in women. The rate of FM loss was attenuated with patients’ age.

**Conclusions:**

Over the period of 12 months following LSG, the reduction of FM was more than twice as much as decrease of LBM. The loss of AbdF was slower than a loss of peripheral subcutaneous fat.

## Introduction

The prevalence of obesity increases dramatically worldwide. According to recent World Health Organization report in the year 2008, approximately 1 billion adults were overweight and around 500 million were obese. Eight years later, more than 1.9 billion persons were overweight and over 650 million were obese [1]. Obesity, especially visceral obesity, is associated with numerous metabolic abnormalities, such as insulin resistance and type 2 diabetes mellitus, chronic inflammation, systemic hypertension, dyslipidemia, endothelial dysfunction, and ischemic heart disease [2–4].

Adiposity can be estimated by simple, anthropometric measures, such as body mass index (BMI), waist and hip circumferences, as well as by much more precise techniques such as CT scans, whole-body magnetic resonance imaging (MRI), and dual-energy X-ray absorptiometry (DXA), which is considered the gold standard in noninvasive assessment of body composition [5]. Whole body DXA scans offer accurate measures of three main body compartments: bone mineral content, fat mass (FM), and lean body mass (LBM), with low radiation exposure [6].

Bariatric surgery has been shown to be the most effective strategy to achieve a significant and long-term weight loss in patients with severe obesity [7, 8]. Laparoscopic sleeve gastrectomy (LSG) and Roux-en-Y gastric bypass (RYGB) are the most commonly performed procedures [9]. The aim of our study was to assess changes in body weight, body composition, and fat distribution measured by DXA, in patients following LSG, over a 12-month period.

## Materials and Methods

A group of 271 obese Caucasian patients (202 women and 69 men), assigned to bariatric surgery by a multidisciplinary team including a nutritionist, psychiatrist, psychologist, internist, and a bariatric surgeon, were included into the study. Exclusion criteria comprised pregnancy, previous bariatric surgery, and secondary obesity, e.g., due to endocrine disorders.

The patients were 18–65 years old, with BMI ≥ 35 or ≥ 30 kg m^−2^ with at least one comorbidity. All patients underwent LSG in compliance with standardized protocols. Prior to the surgery, baseline anthropometric parameters were determined: body weight (BW) was measured with a digital scale to the nearest 0.1 kg and height, with a fixed wall stadiometer, to the nearest 0.1 cm. BMI was calculated and expressed in kilograms per square meter. Excess weight (ExW) was defined as the difference between preoperative weight and ideal body weight based on a BMI of 25 kg m^−2^ and excess weight percentage (ExW%) was calculated according to the formula (ExW / BW) × 100. Waist and hip circumferences (WC, HC) were measured to the nearest 1 cm, and waist-to-hip ratio (WHR) and waist-to-BMI ratio (W/BMI) were calculated.

**Fig. 1:**
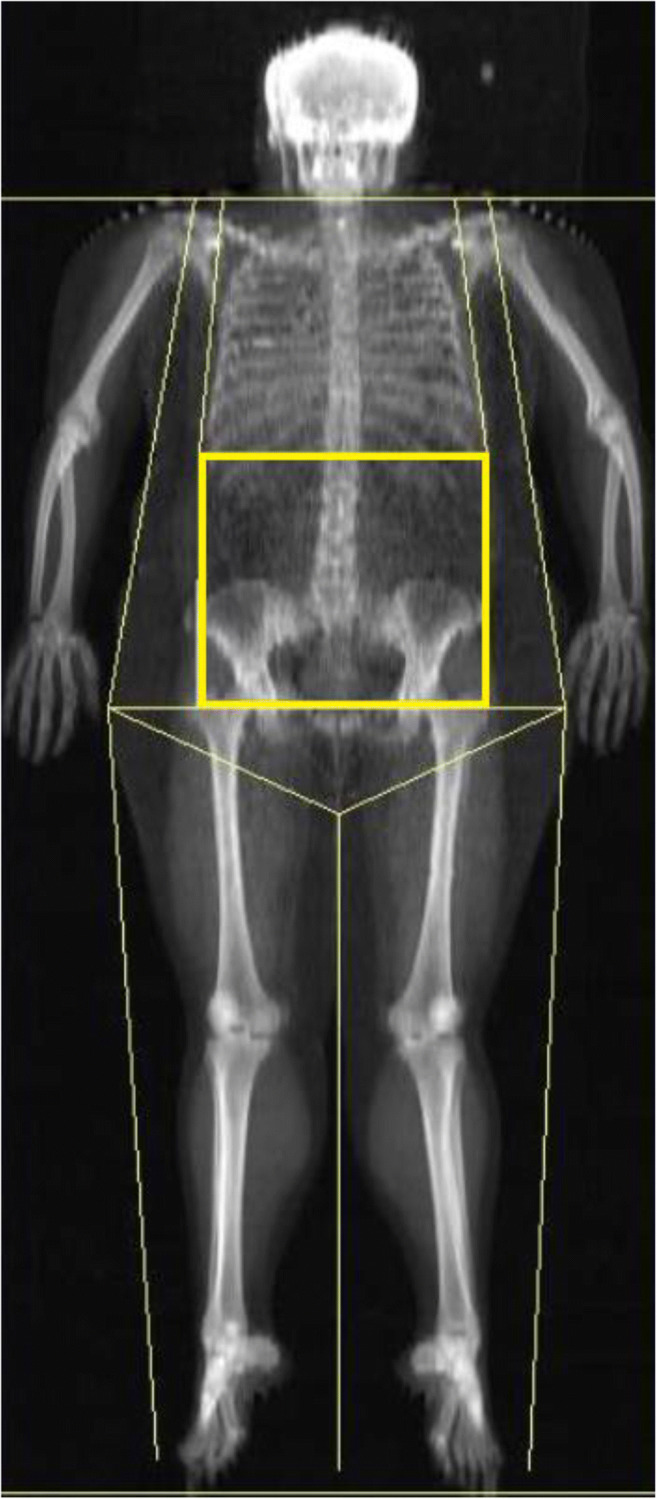
Total body DXA scan with the region of measurement of abdominal fat

Body composition was determined with DXA using HOLOGIC DELPHI system with QDR Software, v.11.1, Hologic Bedford, MA. Whole-body scans were performed by personnel trained and certified in DXA. Daily automatic calibration checks of the system were performed throughout the study with a spine phantom provided by the manufacturer. Whole body FM, body fat percentage (BF%), LBM, and subcutaneous fat (SubF) were determined. Abdominal fat (AbdF) was measured within the region between the top of the pubic bone and extending cranially up to the line between 12th thoracic and 1st lumbar vertebras. The region was laterally enclosed by cutlines between external side of costal arch and femoral great trochanter (Fig. 1). After the scan was completed, abdominal fat to total fat mass ratio (AbdF/FM) was calculated.

Follow-up examinations were performed 12 months after LSG in 155 of the patients (117 women and 38 men). One hundred sixteen patients missed follow-up visit and were excluded from the final analysis. All calculations of weight loss (WL), excess weight loss (ExWL), changes in body composition, and adipose tissue distribution were performed in 155 patients that completed both initial and follow-up examinations. Percentage of excess weight loss (ExWL%) was defined as preoperative weight minus follow-up weight, divided by the excess weight, and multiplied by 100. The percent changes of other values were determined by the equation: 100 × (final − baseline) / baseline.

### Statistical Analysis

The results are presented as mean ± standard deviation (± SD). Normal distribution of continuous variables was assessed with the Shapiro-Wilk test, and homogeneity of variance was determined with Levene’s test. The paired or nonpaired Student’s *t* tests were performed to compare parametric data, and Mann–Whitney *U* test and the Wilcoxon test were used to compare nonparametric variables. For the correlation assessment, the Spearman’s (*r*) coefficient was computed. Multivariable linear regression models were used to investigate the relationship between baseline patients’ characteristics and changes in body weight and composition. Independent variables were categorized according to the quartiles. Stepwise selection in both directions with Akaike’s information criterion was performed to select variables for multivariable models. The calculations were performed using Statistica software, version 13.1. The *p* value < 0.05 was considered significant.

## Results

Basic demographic and anthropometric data as well as body composition of the patients prior to the LSG are presented in Table 1. Out of 155 patients, in 121 (78.1%), BMI was ≥ 40 kg m^−2^, and in 23 (14.8%), BMI was ≥ 50 kg m^−2^.

**Table 1:** Basic characteristics of the patients (*n* = 155) prior to laparoscopic sleeve gastrectomy

	Mean ± SD (range)
Age (years)	42.0 ± 10.5 (18–65)
BW (kg)	124.1 ± 18.4 (80.1–191.7)
ExW (kg)	53.2 ± 15.9 (15.8–106.0)
ExW%	75.2 ± 22.5 (22.1–151.2)
BMI (kg m^−2^)	43.9 ± 5.6 (30.5–62.8)
WC (cm)	126.1 ± 10.6 (104–160)
HC (cm)	134.5 ± 13.7 (115–175)
WHR (cm cm^−1^)	0.94 ± 0.10 (0.72–1.25)
W/BMI (cm kg^−1^ m^−2^)	2.92 ± 0.33 (2.06–3.65)
FM (kg)	54.1 ± 11.1 (22.3–87.7)
BF%	44.5 ± 5.5 (26.1–59.5)
LBM (kg)	64.5 ± 10.6 (45.5–100.9)
SubF (kg)	40.9 ± 9.5 (14.1–64.9)
AbdF (kg)	13.2 ± 3.1 (7.6–25.4)
AbdF/FM (%)	24.9 ± 4.7 (13.5–40.3)

*BW*, body weight; *ExW*, excess weight; *ExW%*, excess weight percentage; *BMI*, body mass index; *WC*, waist circumference; *HC*, hip circumference; *WHR*, waist-to-hip ratio; *W/BMI*, waist-to-BMI ratio; *FM*, fat mass; *BF%*, body fat percentage; *LBM*, lean body mass; *SubF*, subcutaneous fat; *AbdF*, abdominal fat; *AbdF/FM*, abdominal fat to total fat mass ratio

DXA measurements performed before the surgery revealed significant differences in body composition between men and women, in spite of similar BMI in both sexes (Table 2). Males had larger BW (by 12.3%) and LBM (by 29.6%), while women were characterized by higher BF% (by 20.1%) and SubF (by 12.5%). Furthermore, men had higher measures of central adiposity, such as WC (by 10.8%), WHR (by 16.1%), and W/BMI (by 13.5%), as well as larger AbdF (by 20.5%) and AbdF/FM (by 26.9%). To assess the influence of menopausal status on body composition of female patients, the comparison of women aged < 45 years (presumptively premenopausal) and women at the age > 55 years (presumptively postmenopausal) was performed [10]. It was found that whole body fat mass in younger (N-73) and older (N-11) women was 52.9 ± 9.8 vs. 54.3 ± 8.3 kg, *p* = 0.69, respectively, BF% was 45.5 ± 3.7 vs. 47.6 ± 3.6%, *p* = 0.16, respectively, and LBM was 60.1 ± 7.2 vs. 57.2 ± 6.2 kg, *p* = 0.33, respectively. Moreover, both groups of women were characterized by similar values of SubF (40.5 ± 8.2 vs. 41.2 ± 6.2 kg, *p* = 0.82), AbdF (12.4 ± 2.6 vs. 13.1 ± 2.2 kg, *p* = 0.32), and AbdF/FM (23.6 ± 3.6 vs. 24.2 ± 1.6%, *p* = 0.56).

**Table 2:** Basic characteristics of female and male patients prior to laparoscopic sleeve gastrectomy

	Females (*n* = 117)	Males (*n* = 38)	*p* value
Age (years)	41.1 ± 10.5	44.9 ± 10.1	0.06
BW (kg)	120.5 ± 15.6	135.3 ± 21.5	< 0.001
ExW (kg)	52.0 ± 14.6	56.8 ± 19.1	0.10
ExW%	76.2 ± 22.1	72.3 ± 23.5	0.36
BMI (kg m^−2^)	44.1 ± 5.5	43.1 ± 5.9	0.50
WC (cm)	125.3 ± 10.6	138.8 ± 11.1	< 0.001
HC (cm)	135.8 ± 14.6	128.5 ± 9.8	0.07
WHR (cm cm^−1^)	0.93 ± 0.08	1.08 ± 0.08	< 0.001
W/BMI (cm kg^−1^ m^−2^)	2.88 ± 0.27	3.27 ± 0.24	< 0.001
FM (kg)	54.2 ± 10.4	52.2 ± 13.9	0.46
BF%	46.1 ± 4.1	38.4 ± 6.0	< 0.001
LBM (kg)	60.4 ± 7.2	78.3 ± 9.1	< 0.001
SubF (kg)	41.5 ± 8.9	36.9 ± 11.3	0.03
AbdF (kg)	12.7 ± 2.6	15.3 ± 3.6	< 0.001
AbdF/FM (%)	23.8 ± 3.8	30.2 ± 5.3	< 0.001

Data expressed as mean ± SD

*BW*, body weight; *ExW*, excess weight; *ExW%*, excess weight percentage; *BMI*, body mass index; *WC*, waist circumference; *HC*, hip circumference; *WHR*, waist-to-hip ratio; *W/BMI*, waist-to-BMI ratio; *FM*, fat mass; *BF%*, body fat percentage; *LBM*, lean body mass; *SubF*, subcutaneous fat; *AbdF*, abdominal fat; *AbdF/FM*, abdominal fat to total fat mass ratio

Correlations between anthropometric data and body composition of the patients before the surgery were shown in Table 3. It was found that prior to LSG body weight positively correlated with FM, LBM, SubF, and AbdF but not with body fat percentage and AbdF/FM. Anthropometric measures of obesity, such as ExW% and BMI positively correlated with whole body fat mass and percentage, LBM, SubF, and AbdF but negatively correlated with AbdF/FM.

**Table 3:** Correlations between anthropometric characteristics and body composition in patients prior to laparoscopic sleeve gastrectomy

	FM	BF%	LBM	SubF	AbdF	AbdF/FM
BW	0.77***	0.16	0.83***	0.68***	0.69***	− 0.04
ExW%	0.83***	0.45***	0.46***	0.78***	0.58***	− 0.22*
BMI	0.82***	0.44***	0.46***	0.77***	0.56***	− 0.23*
WC	0.46***	− 0.10	0.75***	0.30*	0.68***	0.30*
HC	0.64***	0.50***	0.19	0.57***	0.39**	− 0.13
WHR	− 0.21	− 0.55***	0.46***	− 0.26*	0.19	0.34**
W/BMI	− 0.61***	− 0.66***	0.17	− 0.58***	−0.03	0.43***

*BW*, body weight; *ExW%*, excess weight percentage; *BMI*, body mass index; *WC*, waist circumference; *HC*, hip circumference; *WHR*, waist-to-hip ratio; *W/BMI*, waist-to-BMI ratio; *FM*, fat mass; *BF%*, body fat percentage; *LBM*, lean body mass; *SubF*, subcutaneous fat; *AbdF*, abdominal fat; *AbdF/FM*, abdominal fat to total fat mass ratio

*p < 0.05; **p < 0.01; ***p < 0.001

Correlations between measures of central adiposity and body composition were not uniform. Waist circumference positively correlated with FM, LBM, SubF, AbdF, and AbdF/FM. Waist-to-hip ratio positively correlated with LBM and AbdF/FM but negatively correlated with BF% and SubF. Waist-to-BMI ratio positively correlated with AbdF/FM but negatively correlated with FM, BF%, and SubF. Furthermore, HC correlated positively with total fat mass and percentage as well as SubF and AbdF.

Changes in anthropometric parameters and body composition following LSG were shown in Table 4. It was found that over a 12-month period after surgery, patients’ weight decreased by 28.5 ± 8.7%, ExW by 66.5 ± 22.4%, and BMI by 28.2 ± 9.0%. In 122 (78.7%) patients, ExWL was greater than 50%, and in 65 patients (41.9%), it was greater than 75%. The reduction of BW was the result of a decrease in FM by 44.2 ± 14.1% and LBM by 16.3 ± 5.4%. The BF% decreased after surgery by 22.9 ± 14.9%.

**Table 4:** Changes in anthropometric parameters and body composition within 12 months following laparoscopic sleeve gastrectomy

	Before LSG	After LSG	Mean difference	Difference range	*p* value
BW (kg)	124.1 ± 18.4	88.7 ± 17.0	− 35.4 ± 12.6	− 81.7 ÷ − 8.7	< 0.001
ExW (kg)	53.2 ± 15.9	17.8 ± 15.2	− 35.4 ± 12.6	− 81.7 ÷ − 8.7	< 0.001
ExW%	75.2 ± 22.5	25.1 ± 21.4	− 50.1 ± 22.4	− 119.1 ÷ − 12.1	< 0.001
BMI (kg m^−2^)	43.9 ± 5.6	31.4 ± 5.4	− 12.5 ± 4.4	− 23.5 ÷ − 2.0	< 0.001
WC (cm)	126.1 ± 10.6	98.5 ± 11.9	− 27.6 ± 9.6	− 48.0 ÷ − 3.0	< 0.001
HC (cm)	134.5 ± 13.7	109.7 ± 11.7	− 24.8 ± 10.7	− 56.0 ÷ − 4.0	< 0.001
WHR (cm cm^−1^)	0.94 ± 0.10	0.90 ± 0.08	− 0.04 ± 0.08	− 0.22 ÷ 0.16	< 0.001
W/BMI (cm kg^−1^ m^−2^)	2.92 ± 0.33	3.21 ± 0.39	+ 0.29 ± 0.27	− 0.22 ÷ 1.38	< 0.001
FM (kg)	54.1 ± 11.1	30.2 ± 10.4	− 23.9 ± 8.9	− 57.7 ÷ − 4.0	< 0.001
BF%	44.5 ± 5.5	34.3 ± 7.5	− 10.2 ± 5.3	− 21.3 ÷ 0.5	< 0.001
LBM (kg)	64.5 ± 10.6	54.0 ± 9.7	− 10.5 ± 3.8	− 20.6 ÷ − 3.2	< 0.001
SubF (kg)	40.9 ± 9.5	22.0 ± 8.4	− 18.9 ± 7.5	− 42.5 ÷ − 3.8	< 0.001
AbdF (kg)	13.2 ± 3.1	8.2 ± 2.7	− 5.0 ± 2.7	− 15.2 ÷ 0.1	< 0.001
AbdF/FM (%)	24.9 ± 4.7	28.0 ± 5.8	+ 3.1 ± 5.4	− 10.5 ÷ 20.7	< 0.001

Data expressed as mean ± SD

*BW*, body weight; *ExW*, excess weight; *ExW%*, excess weight percentage; *BMI*, body mass index; *WC*, waist circumference; *HC*, hip circumference; *WHR*, waist-to-hip ratio; *W/BMI*, waist-to-BMI ratio; *FM*, fat mass; *BF%*, body fat percentage; *LBM*, lean body mass; *SubF*, subcutaneous fat; *AbdF*, abdominal fat; *AbdF/FM*, abdominal fat to total fat mass ratio

Waist and hip circumferences decreased by 21.9 ± 7.3% and by 18.4 ± 6.9%, respectively, and WHR by 18.4 ± 6.9%. Subcutaneous fat decreased by 46.3 ± 15.2%, abdominal fat mass decreased by 37.9 ± 16.8%, whereas AbdF/FM and WC/BMI increased by 12.5 ± 23.7 and 9.9 ± 10.1%, respectively.

Changes in all determined parameters were similar in men and women except a more pronounced reduction of WHR and AbdF in men (Table 5).

**Table 5:** Changes in anthropometric parameters and body composition within 12 months following laparoscopic sleeve gastrectomy in females and males

	Females (*n* = 117)	Males (*n* = 38)	*p* value
BW (kg)	− 35.1 ± 10.4	− 36.3 ± 18.0	0.85
ExW (kg)	− 35.1 ± 10.4	− 36.3 ± 18.0	0.63
ExW%	− 51.4 ± 15.1	− 46.2 ± 22.4	0.11
BMI (kg m^−2^)	− 12.7 ± 3.9	− 11.3 ± 5.5	0.09
WC (cm)	− 26.5 ± 9.2	− 33.0 ± 10.2	0.10
HC (cm)	− 26.6 ± 10.9	− 21.0 ± 8.9	0.33
WHR (cm cm^−1^)	− 0.03 ± 0.08	− 1.00 ± 0.08	0.01
W/BMI (cm kg^−1^ m^−2^)	0.30 ± 0.27	0.26 ± 0.30	0.60
FM (kg)	− 23.6 ± 7.6	− 25.0 ± 12.7	0.81
BF%	− 9.9 ± 4.9	− 11.5 ± 6.5	0.28
LBM (kg)	− 10.3 ± 3.6	− 11.0 ± 4.6	0.45
SubF (kg)	− 19.1 ± 6.7	− 18.3 ± 10.2	0.70
AbdF (kg)	− 4.6 ± 2.0	− 6.7 ± 3.9	0.01
AbdF/FM (%)	3.6 ± 4.9	1.9 ± 7.3	0.20

Data expressed as mean ± SD

*BW*, body weight; *ExW*, excess weight; *ExW%*, excess weight percentage; *BMI*, body mass index; *WC*, waist circumference; *HC*, hip circumference; *WHR*, waist-to-hip ratio; *W/BMI*, waist-to-BMI ratio; *FM*, fat mass; *BF%*, body fat percentage; *LBM*, lean body mass; *SubF*, subcutaneous fat; *AbdF*, abdominal fat; *AbdF/FM*, abdominal fat to total fat mass ratio

The associations of baseline variables and changes in body weight and composition following laparoscopic sleeve gastrectomy are shown in Table 6. The stepwise linear regression analysis (with age, BW, BMI, FM, BF%, SubF, AbdF, and AbdF/FM tested for inclusion in the multivariable models) revealed negative associations between patients’ age before surgery and changes of BW, FM, and LBM following LSG. It was found that patients aged 35–41 years had over 7 kg lower reduction of body weight and over 4 kg lower reduction of fat mass compared with patients 18–34 years old (β, − 7.07 (CI, − 12.80 ÷ − 1.34) and β, − 4.77 (CI, − 9.18 ÷ − 0.36), respectively), while patients aged 49–65 years had over 9 kg lower reduction of BW and over 6 kg lower reduction of FM compared with patients 18–34 years old (β, − 9.25 (CI, − 14.57 ÷ − 3.93) and β, − 6.55 (CI − 10.61 ÷ − 2.49), respectively). On the other hand, increased difference in AbdF/FM was found in patients aged 42–48 years compared with persons aged 18–34 years.

**Table 6:** Multiple stepwise regression analyses showing associations of baseline variables and changes in body weight and composition within 12 months following laparoscopic sleeve gastrectomy

	ΔBW		ΔFM		ΔLBM		ΔAbdF/FM	
	β	95% CI	β	95% CI	β	95% CI	β	95% CI
Age (years; ref., 18–34)								
35–41	− 7.07*	− 12.80 ÷ − 1.34	− 4.77*	− 9.18 ÷ − 0.36	− 2.91**	− 4.92 ÷ − 0.90	1.07	− 1.66 ÷ 3.80
42–48	− 7.30**	− 12.45 ÷ − 2.15	− 6.31**	− 10.30 ÷ − 2.31	− 1.68	− 3.44 ÷ 0.08	4.09**	1.68 ÷ 6.49
49–65	− 9.25***	− 14.57 ÷ − 3.93	− 6.55**	− 10.61 ÷ − 2.49	− 2.12*	− 3.97 ÷ − 0.27	2.32	− 0.17 ÷ 4.82
BW (kg; ref., 80.1–110.6)								
110.7–123.6	9.89***	4.52 ÷ 15.25						
123.7–133.4	7.89**	2.37 ÷ 13.40						
133.5–191.7	14.09***	8.68 ÷ 19.50						
BMI (kg m^−2^; ref., 30.5–40.3)								
40.4–43.1					0.06	− 2.13 ÷ 2.25		
43.2–46.6					1.51	− 0.90 ÷ 3.92		
46.7–62.8					3.69*	0.52 ÷ 6.85		
FM (kg; ref., 22.3–44.5)								
44.6–53.3			2.93	− 1.32 ÷ 7.18	− 0.90	− 3.24 ÷ 1.44	− 2.98*	− 5.70 ÷ − 0.26
53.4–60.3			7.92***	3.73 ÷ 12.11	− 2.16	− 5.01 ÷ 0.69	− 5.15***	− 8.05 ÷ − 2.24
60.4–87.7			8.86***	4.66 ÷ 13.07	− 5.07**	− 8.67 ÷ − 1.46	− 5.75***	− 8.76 ÷ − 2.74
LBM (kg; ref., 45.5–56.9)								
57.0–62.5					1.43	− 0.48 ÷ 3.34		
62.6–71.0					1.44	− 0.53 ÷ 3.41		
71.1–100.9					4.13***	1.73 ÷ 6.56		
AbdF (kg; ref.,7.6–11.2)								
11.3–13.2							3.23*	0.45 ÷ 6.01
13.3–14.8							3.23*	0.33 ÷ 6.12
14.9–25.4							7.35***	4.31 ÷ 10.39

Δ calculated as (final − initial values)

*BW*, body weight; *BMI*, body mass index; *FM*, fat mass; *LBM*, lean body mass; *AbdF*, abdominal fat; *AbdF/FM*, abdominal fat to total fat mass ratio

*p < 0.05; **p < 0.01; ***p < 0.001, compared with reference values

Significant positive associations were shown between body weight prior to surgery and decrease of BW after LSG, between FM before the operation and reduction of FM following LSG as well as between AbdF prior to the surgery and decrease of AbdF/FM after LSG. It means, i.e., that patients with body weight 110.7–123.6 kg prior to the surgery had over 9 kg larger BW loss, and patients with body weight ≥ 133.5 kg over 14 kg larger reduction of BW following LSG, compared with patients at lowest quartile of BW before surgery (β, 9.89 (CI, 4.52 ÷ 15.25) and β, 14.09 (CI, 8.68 ÷ 19.50), respectively). Moreover, patients at highest quartiles of BMI (≥ 46.7 kg m^−2^) and LBM (≥ 71.1 kg) before surgery were characterized by increased postoperative LBM loss, whereas patients at highest quartile of FM (≥ 60.4 kg) by decreased LBM loss following surgery, compared with patients at lowest quartiles of BMI, LBM, and FM, respectively. Significant negative association was also shown between AbdF prior to operation and changes in AbdF/FM following surgery.

## Discussion

The study aimed to determine the influence of LSG on body composition and fat distribution over the period of 12 months following surgery. Whole body DXA scans were used for precise measurements of total fat mass and percentage, LBM as well as SubF and AbdF. Recently, DXA technology significantly improved the accuracy in the assessment of body composition and made possible the direct measurement of visceral adipose tissue in patients with obesity [6, 11]. With this new technique, visceral fat analysis is performed typically in the android region, which is a small portion of the abdomen included between the line joining the two superior iliac crests and extending cranially up to the 20% of the distance between this line and the base of the skull. In our study, abdominal fat was measured in the larger region extending from pubic bone up to the line between the 12th thoracic and 1st lumbar vertebras, and laterally enclosed by cutlines between the external side of costal arch and femoral great trochanter. This region included both visceral fat and subcutaneous fat localized at anterior and posterior abdominal walls but excluded subcutaneous adipose tissue at lateral parts of the trunk. It was documented that subcutaneous abdominal fat had as strong an association with insulin resistance as visceral fat in obesity [12].

DXA measurements performed in our study revealed that BMI in obese persons did not reflect body composition. It was shown that males and females of similar BMI had different proportions of FM and LBM with bigger lean body mass and AbdF in men and higher BF% in women.

Significant sex differences in body composition have been well documented [13, 14]. It was found that premenopausal women are characterized by higher body fat percentage compared with men with an increased propensity to store adipose tissue in subcutaneous sites, especially in gluteofemoral locations. Estrogen that is a main regulator of body adiposity and fat distribution in women, through activation of estrogen receptor α, promotes energy expenditure and reduces food intake by decreasing expression or release orexigenic peptides such as neuropeptide Y and ghrelin [15]. The loss of estrogens with menopause is associated with an increase in intra-abdominal fat accrual and somewhat smaller loss in lean body mass [16]. Men, due to anabolic and adipose-regulating effects of testosterone are characterized by higher lean mass and abdominal adiposity [13, 17].

In our study, hormonal status of female patients was not determined. Like Ochner et al. [10], we considered women at the age of < 45 years as premenopausal and women > 55 years old as presumptively postmenopausal. No significant differences were found in body composition and fat distribution between younger and older women, but the number of patients, especially in older group was very small.

The analysis of correlations between preoperative anthropometric and densitometric measurements showed that in patients with obesity, both FM and LBM increased together with BMI value, but the growth rate of abdominal adipose tissue was slower than that of peripheral, subcutaneous fat. It should be stressed that anthropometric parameters of central obesity, especially W/BMI, significantly correlated with AbdF/FM and could be considered a reliable measure of abdominal fat tissue.

The comparison of measurements performed before and after bariatric surgery showed that within 12 months following LSG mean patients’ weight decreased by 28.5%, ExW by 66.5%, and BMI by 28.2%, with great heterogeneity at an individual level. Therapeutic success rate (ExWL ≥ 50%) was attained in 78.7% of patients. It is of significance that reduction of FM (− 23.9 kg) was more than twice as much as decrease of LBM (− 10.5 kg). Relative preservation of LBM (mainly muscle mass) after bariatric surgery is particularly important as it is involved in maintenance of patients’ functional and physical capacities [18, 19].

Few studies reported changes in body composition after bariatric surgery assessed by DXA. Bazzocchi et al. examined 29 women before and after RYGB and found that FM and LBM decreased by 50.9 and 9.6%, respectively, over a 12-month period [20]. Kenngott et al., using MRI revealed that over the period of 12 months following surgery, the volumes of subcutaneous and visceral adipose tissues went down by 42.3 and 52.3%, respectively, and skeletal muscle volume by 11.1%, in 31 patients with LSG (N-20) or RYGB (N-11) [21]. Kavanagh et al. analyzed the results of measurements of total body mass, lean mass, and fat mass by air displacement plethysmography in 63 patients undergoing either LSG (N-33) or RYGB (N-30). They found that within 12 months following surgery, mean ExWL in the LSG and RYGB groups was 47.2 and 53.4%, respectively, mean percent change of fat mass was 9.2 and 10.5%, respectively, and mean percent change of lean body mass was 9.4 and 10.5%, respectively [22].

The analysis of DXA scans performed in our study indicated that the loss of AbdF over the period of 12 months following LSG was slower than a decrease of peripheral subcutaneous fat. It was found that SubF decreased by 46.3 ± 15.2%, AbdF decreased by 37.9% after surgery, as a result AbdF/FM increased significantly by 12.5%, together with W/BMI that increased by 9.9%.

The study showed that together with patients’ age the rate of post-operative BW loss and FM loss was attenuated. Furthermore, the rates of fat and lean mass loss were higher in patients with more severe obesity, while decrease of abdominal fat was more pronounced in patients with central adiposity.

In the previous studies a higher relative reduction of visceral fat as compared to other compartments was observed [23–26]. The authors of Swedish study examined body composition of 166 women before and 2 years after RYGB. They found that visceral fat to total fat mass ratio, as well as android to gynoid fat mass ratio decreased, suggesting that visceral adipose tissue within the android region, determined by DXA software in the upper part of the abdomen, is more mobilized than subcutaneous fat depots [23]. Maïmoun et al. showed that within 12 months following LSG, abdominal visceral fat, measured with DXA in 30 obese patients, decreased by 44.6%, while abdominal subcutaneous adipose tissue decreased by 39.7% [24]. Hui et al. measured the change in fat content in a group of 12 volunteers with morbid obesity before and after bariatric surgery (8 of them after LSG) using MRI whole-body scanner. The MRI acquisition for abdominal subcutaneous and visceral adipose tissue measurements was performed from the dome of the diaphragm to the pubic symphysis. The authors found that within 12 months after surgery, subcutaneous adipose tissue volume decreased by 34.5% and visceral adipose tissue volume by 51.2%, mostly in the first 6 months following surgery [25]. The study using whole-body MRI showed that relative loss was significantly higher for visceral than for subcutaneous adipose tissue (52.3 vs. 42.3%) 12 months following bariatric surgery [21]. Meta-analysis made by Merlotti et al., comprising 89 studies, documented that in patients that reduced BW with different strategies (diet and/or physical activity, weight-loss promoting drugs and bariatric surgery), the percentage loss of visceral fat was always greater than percentage loss of subcutaneous adipose tissue [26].

On the other hand, the authors of the Swiss study, which included 16 patients with morbid obesity (4 scheduled for LSG and 12 for RYGB), showed, that both visceral and subcutaneous fat, measured with MRI scanning, decreased with similar rate, up to 24 months after bariatric surgery [27]. Bazzocchi et al. also found that visceral and subcutaneous abdominal adipose tissues had been reduced with similar rate, by 65.6 and 57.9%, respectively, in women over a period of 12 months following RYGB [20]. Meta-analysis of 61 studies made by Chaston et al. suggested that visceral adipose tissue was lost preferentially with modest weight loss, but with greater weight loss, this effect was attenuated [28].

Unlike this observation, Wu et al. found, that in 25 patients after bariatric surgery with BMI loss of 16.0%, abdominal visceral and abdominal subcutaneous fat volumes, measured with CT, decreased by 40.5 and 31.4% respectively, while in 39 patients with BMI loss of 7.5% with exercise, the reduction of visceral and subcutaneous adipose tissue was 15.2 and 17.3%, respectively [29].

Further studies are needed to elucidate the observed divergences and to better understand the mechanisms that influence changes in body composition and fat distribution after bariatric surgery.

## Conclusion

The results of our study showed that the reduction of whole body fat mass was more than twice as much as decrease of lean body mass in patients with obesity within 12 months after LSG. The loss of abdominal adipose tissue was slower than the reduction of peripheral subcutaneous fat, and the loss of abdominal adipose tissue was more pronounced in men than in women.
